# 70th Year Anniversary of Carbon Nanotube Discovery—Focus on Real-World Solutions

**DOI:** 10.3390/nano13243162

**Published:** 2023-12-18

**Authors:** Muralidharan Paramsothy

**Affiliations:** NanoWorld Innovations (NWI), 1 Jalan Mawar, Singapore 368931, Singapore; mpsothy@yahoo.co.uk

Seventy years ago in 1952, Russian scientists LV Radushkevich and VM Lukyanovich published clear images showing multiwalled carbon nanotubes (MWCNTs) with 50 nm diameters [1]. Their paper was written in Russian and published in *Zhurnal Fizicheskoi Khimii* (now the Russian Journal of Physical Chemistry A) at the height of the Cold War. It is noteworthy that S Iijima’s work, published some 30 years ago in 1991, also generated unprecedented interest in carbon nanostructures, including CNTs, and has since fueled intense research in the area of nanotechnology [2]. *Radushkevich and Lukyanovich should be credited for the discovery that carbon filaments can be hollow and have a nanometer-size diameter, i.e., for the discovery of CNTs* [3].

CNTs are recognized for ultrahigh strength and deformability, high thermal conductivity, ballistic electrical conductivity, selected biocompatibility, unusual optical properties, and high surface area. Graphene and nanohorns are other well-known nanoscale forms of carbon, but CNTs remain distinguished by virtue of their one-dimensional or 1D structure, enabling directional tailoring of exceptionally favorable characteristics (enabling desired anisotropic properties) when required in application. Advancing the contemporary theme of sustainability, 14 representative articles have been published in this Special Issue. Of these, 3 articles highlight nanoscale fundamental aspects of CNTs, i.e., diameter measurement [4], rotor system molecular simulation [5] and ultra-high tensile strength [6]. Eleven articles are on applications utilizing CNTs pertaining to energy [7,8], filtration via membrane distillation [9], environmental remediation using adsorption [10], ionic liquids as curing agents [11], biosensors [12] and bioinspired sensors [13], and electrical/mechanical properties of polymer nanocomposites [14,15,16,17]. Geographically speaking, including in terms of mixed-country authorship, this Special Issue is of a truly global nature: 3 articles are from Canada, 1 article is from Saudi Arabia, 3 articles are from China, 2 articles are from Russia, 2 articles are from Spain, 3 articles are from India, 1 article is from Japan, 1 article is from United Arab Emirates (UAE), 2 articles are from USA, 1 article is from Germany, 1 article is from Turkiye, and 1 article is from Malaysia, with the Guest Editor being from Singapore, and who has published critical work on the CNT-polymer interface towards mechanical properties of the nanocomposite, up to 20 years ago [18,19].

## Diameter Measurement

*Making a valuable contribution to nanoscale research*, Lopinski et al. [4] developed a method using atomic force microscopy (AFM) for reliable and accurate diameter measurements on ‘real-world’ supported samples, such as those used for device fabrication. To illustrate the utility of this diameter measurement method, it was applied to measure the diameter of commercially available, highly enriched semiconducting nanotubes. The measured diameter for the nanotubes in a poly(9,9-di-n-dodecylfluorene) single-wall CNT (PFDD/SWCNT) sample was shown to be larger than expected based on the diameters of the tubes present in the dispersion, suggesting the polymer remained on the SWCNTs upon deposition onto the substrate and rinsing with a solvent. The average thickness of the polymer on the nanotubes was estimated to be ~0.4 nm. In addition, the measured heights of tube–tube junctions were found to be smaller than the diameters of the two tubes that cross, indicating a compression of ~20% at junctions. *Finally, it was noted that the measurement protocol developed should also be generally applicable to the measurement of vertical dimensions of other 1D or 2D nanomaterials.*

## Rotor Systems Molecular Simulation

*Undertaking fundamental exploration*, Qing Peng et al. [5] investigated the rotating and braking processes of a carbon nanotube transmission system using simulations of molecular dynamics. The effect of hydroxyl groups on the speed of response was examined. The energy dissipation during the whole process was discussed. The results showed that hydroxyl groups enhanced stability and reduced the response time of the system in both the acceleration and braking processes. The higher hydroxyl group ratio enabled the achievement of a better transmission system performance. The underlying mechanism was the presence of hydrogen bonds that form between hydroxyl groups, which resulted in higher interfacial interaction and a faster response. The analysis of the phonon density of the state showed that the vibration of O-H bonds in hydroxyl groups accelerated the energy dissipation of the system, which led to faster responses in the acceleration and braking processes. *The results showed that grafted hydroxyl groups resulted in stronger interaction, and therefore had the potential to enhance the response of the transmission system.*

## Ultra-High Tensile Strength

*In a critical review*, Fei Wei et al. [6] analyzed the controllable preparation and tensile strength of CNTs at different scales. Operating based on recent years’ research into the controllable preparation of CNT fibers (CNTFs), the significance of defect control and efficient treatment process to control tube–tube interactions was emphasized in research towards the development of ultra-high-strength CNTFs. The unique structure and large-scale preparation of CNTs, which constitute the basic unit of nanoscale assembly, was introduced. It was observed that the mechanical strength and toughness could be significantly improved by eliminating the tube–tube non-uniform interactions or other post-treatment interactions. Using the bottom-up method, the mass production of defect-free CNTs and their accurate assembly into the macro-scale products with fewer defects, fine alignment, and higher density was the priority, with the eventual aim of obtaining macro-scale assemblies with excellent tensile strength. Not only tensile strength, but also other mechanical properties such as fatigue, bending, and torsion, as well as electrical and thermal properties, all faced a similar problem of performance transfer across scales. *It was also observed that, with further analysis of the influencing factors and optimization strategies for the cross-scale transfer of other properties, including overcoming selected challenges, the excellent intrinsic properties of CNTs could be fully utilized, and CNTs could play a more significant role in many fields.*

## Energy

*Groundbreakingly*, Bulusheva et al. [7] synthesized composite nanomaterials via the vaporization–condensation process using commercial red phosphorus and single-walled CNTs (SWCNTs). Under the synthesis conditions in play, phosphorus chains were formed inside open SWCNTs and the SWCNT surface was covered with red phosphorus, which was oxidized upon contact with air. The external phosphorus deposit was effectively removed using an aqueous solution of sodium hydroxide. The content of phosphorus in samples with only external phosphorus SWCNTs/P, only internal phosphorus P@SWCNTs and both types of phosphorus P@SWCNTs/P was 11%, 8% and 16%, respectively, according to XPS data. To reveal the effect of various combinations of SWCNTs and phosphorus in the composite on the electrochemical interaction with lithium ions, nanomaterials were tested as anodes in coin-cell batteries using lithium sheets as counter electrodes. The SWCNTs/P sample was found to perform better than the reference red phosphorus due to the presence of a conducting network of SWCNTs. Nanotubes practically did not contribute to the SWCNTs/P capacity because their surface was blocked by phosphorus. The creation of composite nanomaterials with internal phosphorus almost doubled the initial Coulombic efficiency as compared to SWCNTs/P and dramatically improved the specific capacity and cycling stability of the electrode. The presence of both external and internal phosphorus led to a gradual and slight degradation of the P@SWCNTs/P electrode over time, associated with slower diffusion processes in the internal volume of the material. *The excellent cycling stability of P@SWCNTs over 1000 cycles at a high current density of 5 A·g−1 was associated with the synergistic effect of highly capacitive phosphorus and conductive SWCNTs, the absence of inactive oxidized phosphorus deposits, and the presence of multiple channels for lithium-ion diffusion to encapsulated phosphorus.*

*Uniquely*, Archana et al. [8] successfully synthesized titanium dioxide/reduced graphene oxide/silver (TiO_2_/rGO/Ag) hybrid nanostructures using a combination of solution processes and in situ growth. These hybrid nanostructures were then utilized as photoanodes for dye-sensitized solar cells (DSSCs) and as catalysts for photodegradation applications. Plasmon-enhanced DSSC devices demonstrated enhanced photovoltaic performance of 7.27% along with a higher short-circuit current of 16.05 mA/cm^2^ and an incident photocurrent efficiency (IPCE) of 77.82% at 550 nm. The results suggested that the high photovoltaic performance of the plasmon-based TiO_2_/rGO/Ag device could be attributed to (i) the large specific area of TiO_2_/rGO/Ag, which led to high dye loading; (ii) TiO_2_ mesospheres enhancing the light scattering effect of incoming light; and (iii) the incorporation of Ag nanoparticles (NPs) facilitating more induced photons and fast electron transport in the device. Upon natural sunlight irradiation, the prepared hybrid nanostructure showed a 93% improvement in the photocatalytic degradation of methylene blue (MB) dye within 160 min, and the effects of different scavengers on the obtained photocatalytic activity were systematically investigated. The effects of the optimum active surface area, the localized surface plasmon resonance (LSPR) properties of Ag NPs, and the enhanced electrical conductivity of the prepared ternary nanostructures combined to provide an enhanced visible-light-driven plasmonic DSSC device and photocatalyst for MB dye degradation. *The proposed plasmonic and hybrid-based nanostructures demonstrated an emerging strategy to establish large-scale applications of solar energy conversion technologies.*

## Filtration via Membrane Distillation

*Very relevantly*, Hilal et al. [9] demonstrated that in membrane distillation (MD), the membrane characteristics could be tuned using an electrospray deposition technique to obtain desirable MD membrane properties, such as high hydrophobicity or high water contact angle (>120°), high liquid entry pressure (LEP), optimum pore size (~0.2 µm), narrow pore size distribution, etc., compared to the pristine electrospun membrane. CNT modification followed by heat pressing yielded mechanically robust nanocomposite membranes with improved membrane characteristics. A 3% increase in the water contact angle, 20% increase in the LEP, and 42.6% reduction in the mean flow pore size towards the optimum pore size were observed in the heat-pressed CNT-modified electrospun poly(vinylidene fluoride-co-hexafluoropropylene) (PVDF-Co-HFP) membranes. The tensile strength of the heat-pressed CNT-modified membrane was significantly improved by up to 120% compared to the electrospun PVDF-Co-HFP membrane. The presence of CNTs on the membrane surface before and after the MD process was observed. Water vapor flux enhancements of 15.7%, 20.6%, and 24.6% were observed at a ΔT of 20 °C, 30 °C, and 40 °C, respectively. Higher temperature polarization coefficient (TPC) values and percentage water vapor flux enhancements were observed at lower feed solution temperatures because of the higher heat loss at higher feed solution temperatures compared to the lower temperatures. Enhancements of 16% and 12% in the TPC values were observed at the feed solution temperatures of 35 °C and 55 °C, respectively. A > 99.8% inorganic salt rejection was observed through the use of quantitative analytical tools when conducting the direct contact membrane distillation (DCMD) process using a 3.5 wt. % simulated seawater feed solution. *Electrohydrodynamic atomization using appropriate nanomaterial dispersion can be recommended as an efficient tool for the surface modification of MD membranes.*

## Environmental Remediation Using Adsorption

*Futuristically,* Neelgund et al. [10] successfully prepared an efficient adsorbent for the effective adsorption of Pb(II) and As(III) ions by grafting fourth-generation aromatic poly(amidoamine) (PAMAM) to CNTs and via the successive deposition of Ag nanoparticles. Thus, CNTs-PAMAM-Ag was able to adsorb 99% and 76% of Pb(II) and As(III) ions, respectively, within 15 min. The kinetics data obtained for the adsorption of Pb(II) and As(III) ions were well fitted with the pseudo-second-order model compared to the pseudo-first-order model. This revealed the occurrence of chemisorption due to sharing or exchanging electrons between Pb(II) or As(III) ions and CNTs-PAMAM-Ag. This could be the rate-controlling step in the process of adsorption. The multilinearity of the Weber–Morris plot demonstrated that intraparticle diffusion was not a rate-controlling step in the adsorption of Pb(II) and As(III) ions; instead, it was regulated by both intraparticle diffusion and the boundary layer effect. The proper fitting of kinetic data of Pb(II) and As(III) ion adsorption with the Langmuir isotherm model indicated the uniform distribution of active sites over the entire surface of CNTs-PAMAM-Ag and their homogeneity. In addition, it signified that the adsorption of Pb(II) and As(III) ions was dominated by monolayer binding on the homogeneous surface of CNTs-PAMAM-Ag. The adsorption ability of CNTs-PAMAM-Ag depended on the pH. *The CNTs-PAMAM-Ag was an ideal adsorbent for repeated use without losing its activity, and because of its significance in Pb(II) and As(III) ion adsorption, CNTs-PAMAM-Ag could be an efficient adsorbent with practically applicability for the adsorption of other heavy metals and other contaminants present in water.*

## Ionic Liquids as Curing Agents

*In a green approach*, Guerrica-Echevarría et al. [11] showed the double role of ionic liquids (ILs) as effective curing and dispersing agents in the production of volatile amine-free epoxy/CNT nanocomposites with a better balance of mechanical, electrical, and adhesive properties. Three different ILs were tested, and all three generated good dispersion of the nanofiller, featuring individually dispersed CNTs as well as some small aggregates. Overall, with a percolation threshold of 0.001 wt.%, the IL trihexyltetradecylphosphonium dicyanamide (IL-P-DCA) system was the most effective. The addition of CNTs had no effect on the thermal or low-strain mechanical properties of the epoxy/IL systems. However, CNT addition improved the systems’ adhesive properties. The lap shear strength of epoxy/IL-P-DCA system containing 0.025 wt.% CNTs was improved by 30% compared to that of epoxy/IL-P-DCA. This was the best improvement among the three cases. *This research proved that, using very small amounts of CNTs, it is possible to obtain electrically conductive, amine-free epoxy adhesives with similar mechanical properties but greater lap shear strength than the reference amine-cured epoxy system. Additionally, since ILs have a lower vapor pressure, and a significantly lower amount is needed to effectively cure epoxy resins, we therefore observed that replacing conventional epoxy resin curing agents (amines, anhydrides, etc.) with ILs was a major step forward in the development of more sustainable materials.*

## Biosensors

*Most relevantly and impactfully*, Hussain et al. [12] summarized the advancements in CNT-based biosensors since the last decade in the detection of different human viruses, namely, SARS-CoV-2, dengue, influenza, human immunodeficiency virus (HIV), and hepatitis. It has been proven that viral infections pose a serious hazard to humans and also affect social health, including morbidity and mental suffering, as illustrated by the COVID-19 pandemic. The early detection and isolation of virally infected people are thus required to control the spread of viruses. *Due to the outstanding and unparalleled properties of nanomaterials, numerous biosensors have been developed for the early detection of viral diseases* via *sensitive, minimally invasive, and simple procedures. To that end, viral detection technologies based on CNTs have been developed as viable alternatives to existing diagnostic approaches, and the shortcomings and benefits of CNT-based biosensors for the detection of viruses have also been outlined and discussed.*

## Bioinspired Sensors

*Making practical and logical adoptions from natural biology*, Wu et al. [13] proposed a sensitive capacitive pressure sensor with a broad detection range inspired by the skin epidermis. A simple and low-cost fabrication process was proposed for carbon nanotube/polydimethylsiloxane-based (CNT/PDMS-based) spinosum pressure sensors via the use of abrasive paper templates. The spinosum microstructure and doping content of CNTs effectively improved the performance of pressure sensors: high sensitivity (0.25 kPa^−1^), wider pressure range (~500 kPa), fast response time (20 ms) and excellent stability over 10,000 cycles. Also, the effects of the mesh number of abrasive papers and CNT doping content on the sensing property were theoretically analysed via simulations and experiments. Further, a sensor array was manufactured for mapping the spatial distribution of pressure, which showed great potential for intelligent monitoring. *Finally, a new methodology was proposed in order to solve the tire–road contact pressure issue, as well as estimate related parameters by introducing a sensor array to tires. Practically and logically, a bioinspired, cost-effective, broad-range, high-sensitivity, and flexible sensor was fabricated, and a new patch to intelligently monitor the contact pressure of tires was also developed.*

## Electrical/Mechanical Properties of Polymer Nanocomposites

*In further groundbreaking research,* Dimiev et al. (including A. Nasibulin) [14] controlled the permittivity of dielectric composites for numerous applications dealing with matter/electromagnetic radiation interactions. Polymer composites were prepared with a silicone elastomer matrix and Tuball™ SWCNTs using a simple preparation procedure. The as-prepared composites demonstrated record-high dielectric permittivity in both the low-frequency range (102–107 Hz) and in the X-band (8.2–12.4 GHz), significantly exceeding the literature data for such types of composite materials at similar levels of CNT content. Thus, with the 2 wt% filler loading, the permittivity values reached 360 at 106 Hz and >26 in the entire X-band. In similar literature, even the use of conductive polymer hosts and various highly conductive additives did not result in such high permittivity values. *The superior permittivity phenomenon was attributed to specific structural features of the SWCNTs, namely, length and the ability to constitute percolating networks in the polymer matrix in the form of neuron-shaped clusters. The low cost and large production volumes of Tuball™ SWCNTs, as well as the ease of the composite preparation procedure, opened the doors for the production of cost-efficient, low weight and flexible nanocomposites with superior high permittivity.*

*Critically*, Krause et al. [15] prepared poly(methyl methacrylate) (PMMA)/single-walled carbon nanotube (Tuball™ SWCNT) nanocomposites via melt mixing to achieve suitable SWCNT dispersion and distribution with low electrical resistivity, where the SWCNT direct incorporation method was compared with the results of masterbatch dilution. An electrical percolation threshold of 0.05–0.075 wt% was found, the lowest threshold value for melt-mixed PMMA/SWCNT nanocomposites reported so far. The influence of rotation speed and method of SWCNT incorporation into the PMMA matrix on the electrical properties and SWCNT macro dispersion was investigated. It was found that increasing rotation speed improved macro dispersion and electrical conductivity. The results showed that electrically conductive nanocomposites with a low percolation threshold could be prepared by direct incorporation using high rotation speed. The masterbatch approach led to higher resistivity values compared to the direct incorporation of SWCNTs. Also, the thermal behaviour and thermoelectric properties of PMMA/SWCNT nanocomposites were studied. The Seebeck coefficients varied from 35.8 µV/K to 53.4 µV/K for nanocomposites up to 5 wt% SWCNT. It was also observed that the addition of SWCNT increased the glass transition temperature (T_g_) of PMMA by approximately 4 K. *Overall, the Tuball™ SWCNT material was observed to be an effective filler that could be used to obtain conductive nanocomposites with good dispersion and low electrical resistivity via melt-mixing. Also, the thermoelectric measurements indicated that PMMA/SWCNT nanocomposites could be used as a thermoelectric material.*

*Also critically,* Sundararaj et al. [16] investigated the effects of MWCNT concentration, mixing time, and compatibilizer addition on the migration of MWCNTs from the polyethylene (PE) phase to a polyethylene oxide (PEO) phase of a 60:40 PEO/PE blend, and the subsequent impact on electrical and rheological properties. Two-step mixing was used to pre-localize MWCNTs in the less thermodynamically favoured PE phase and observe migration into the thermodynamically favoured PEO phase. It was observed that MWCNTs migrated into the PEO phase as the mixing time increased at all concentrations of MWCNTs used. This migration was also supported by electromagnetic interference shielding effectiveness (EMI SE) and DC conductivity measurements, which showed significant reductions in electrical properties over time, suggesting a disruption of conductive networks as MWCNTs migrated into PEO. PEO/PE 40:60 samples containing 3 vol% MWCNTs showed a high conductivity of 22.1 S/m, which that suggested effective MWCNT networks were present at the onset of mixing. To arrest the migration of MWCNTs into PEO, a PE-graft-maleic anhydride (PEMA) compatibilizer was added to the PEO/PE blend. An improvement in the formation of MWCNT networks along the PEO/PE interface was observed at 5 min of mixing for the compatibilized polymer blend nanocomposite (PBN). Furthermore, major improvements in electrical conductivity (68.7 S/m) were observed. *Comparisons to the poly(vinylidene fluoride)/poly(ethylene) (PVDF/PE) system in previous research suggested that the viscosity of the destination phase, as well as the interfacial surface energies of the blend components, played significant roles in determining whether MWCNTs would successfully migrate across polymer/polymer interfaces or whether they would become trapped at the interface. Migration behaviour was shown to significantly influence the electrical and rheological properties of PBNs.*

*Of interest in a rather over-arching sense as the first article in this SI*, Nurazzi et al. (including R.A. Ilyas and A. Khalina) [17] reviewed the mechanical performance of CNT-reinforced polymer nanocomposites. It was observed that CNTs have excellent chemical and physical properties, making them ideal and promising for reinforcement in polymer nanocomposites. It was acknowledged that the mechanical properties of the CNT/polymer nanocomposites are influenced by the interactions between the nanofillers and the polymer matrices. It was noted that the main challenge is the tendency of the CNTs to agglomerate, resulting in poor dispersion properties, an issue that can cause the performance of the composite structures to deteriorate. Researchers have devised various methods for distributing and orienting CNTs. Further, it was also observed that dispersing a small amount of filler in the polymer matrix enhanced nanocomposite properties. Although numerous CNT nanocomposites have been investigated, consistent progress is still needed to obtain nanocomposites with the best performance. *Several dimensions, such as the amount of CNTs, size of CNTs, spatial distribution and orientation of CNTs, the suitability of surface modification of the CNT, and method of nanocomposite fabrication, all collectively affect the mechanical properties of the nanocomposite. The crucial nature of necessarily finding a collective balance among these multiple parameters for optimum performance of the CNT nanocomposite was duly reflected.*
